# A cross-sectional analysis of dietary protein intake and body composition among Chinese Americans

**DOI:** 10.1017/jns.2018.31

**Published:** 2019-01-30

**Authors:** Collin J. Popp, Jeannette M. Beasley, Stella S. Yi, Lu Hu, Judith Wylie-Rosett

**Affiliations:** 1 Department of Population Health, NYU School of Medicine, New York, NY 10016, USA; 2 Department of Medicine, NYU School of Medicine, New York, NY 10016, USA; 3 Department of Epidemiology and Population Health, Albert Einstein College of Medicine, New York, NY 10461, USA

**Keywords:** Lean body mass, Muscle mass, Percentage body fat, Adiposity, Obesity, %EI, percentage energy intake, BIA, bioelectrical impedance analysis, BW, body weight, CHA, Chinese Americans, FFM, fat-free mass, FFM%, percentage fat-free mass, FFMI, fat-free mass index, FM, fat mass, FM%, percentage fat mass, FMI, fat mass index, PA, physical activity

## Abstract

Favourable body composition has been associated with higher dietary protein intake. However, little is known regarding this relationship in a population of Chinese Americans (CHA), who have lower BMI compared with other populations. The aim of the present study was to assess the relationship between dietary protein intake, fat mass (FM) and fat-free mass (FFM) in CHA. Data were from the Chinese American Cardiovascular Health Assessment (CHA CHA) 2010–2011 (*n* 1707); dietary intake was assessed using an adapted and validated FFQ. Body composition was assessed using bioelectrical impedance analysis. The associations between protein intake (% energy intake) and BMI, percentage FM (FM%), percentage FFM (FFM%), FM index (FMI) and FFM index (FFMI) were examined using multiple linear regression adjusted for age, sex, physical activity, acculturation, total energy intake, sedentary time, smoking status, education, employment and income. There was a significant positive association between dietary protein and BMI (*B* = 0·056, 95 % CI 0·017, 0·104; *P* = 0·005), FM (*B* = 0·106, 95 % CI 0·029, 0·184; *P* = 0·007), FM% (*B* = 0·112, 95 % CI 0·031, 0·194; *P* = 0·007) and FMI (*B* = 0·045, 95 % CI 0·016, 0·073; *P* = 0·002). There was a significant negative association between dietary protein and FFM% (*B* = −0·116, 95 % CI −0·196, −0·036; *P* = 0·004). In conclusion, higher dietary protein intake was associated with higher adiposity; however, absolute FFM and FFMI were not associated with dietary protein intake. Future work examining the relationship between protein source (i.e. animal) and body composition is warranted in this population of CHA.

Chinese Americans (CHA) represent the largest immigrant population in New York City. As of 2017, the CHA population in New York City reached over 628 000 individuals, a 76 % increase over a 17-year period^(^^1^^)^. The rapid growth of CHA draws health concerns as strong correlations exist between the length of US residency and increased prevalence of overweight, obesity and other non-communicable diseases^(^^2^^–^^5^^)^. The acculturative process is further complicated by the rapid increase of obesity and diabetes occurring currently in China^(^^6^^)^, and how these patterns may be affecting newly arrived immigrant populations in the USA. CHA with obesity (≥27·5 kg/m^2^) are at four times greater risk of developing diabetes and high blood pressure compared with normal-BMI CHA^(^^7^^)^.

Body composition and metabolic risk vary across ethnic populations. Compared with Caucasian adults, Chinese adults experience higher odds of co-morbidities for a given BMI after adjusting for age and sex^(^^8^^)^. As such, modified ethnic-specific BMI cut-offs have been devised^(^^9^^)^. Chinese adults, on average, have lower BMI in comparison with Caucasian adults but higher adiposity stores^(^^10^^–^^12^^)^. While BMI is useful for evaluating body weight (BW) of individuals of different height, it falls short of providing an accurate index of body composition. Height-adjusted indexes for both fat mass (FM) and fat-free mass (FFM), called FM index (FMI) and FFM index (FFMI), respectively, provide a valid and more adequate representation of body composition^(^^13^^)^.

A lifestyle factor often promoted as a way to increase FFM is a high-protein diet. The Institutes of Medicine acceptable macronutrient distribution range (AMDR) of 10–35 % of energy from protein covers a broad range of macronutrient needs depending on age, sex and activity level^(^^14^^)^. A higher-protein diet may exist on the higher end (20–25 % total energy intake) of this range. However, protein intake is traditionally defined in relative terms, with the recommended dietary allowance of 0·8 g protein/kg BW^(^^15^^)^. A higher protein range of 1·2–1·8 g/kg BW is not uncommon in weight loss interventions and athletic populations. Protein intake has been found to have a greater thermic effect of food compared with carbohydrates and fats, leading to postprandial increases in energy expenditure^(^^16^^–^^18^^)^. High-protein meals lead to greater satiety, which may lead to subsequent reductions in total energy intake^(^^19^^,^^20^^)^. Furthermore, higher protein intake has been associated with lower BW, BMI and waist circumference^(^^21^^)^. Epidemiological evidence suggests that high protein intake is associated with higher FFM in adults and older adults^(^^22^^–^^24^^)^.

On average, US adults consume roughly 15 % of their energy from dietary protein^(^^25^^,^^26^^)^. However, above-average protein levels (17–19 % of energy intake) of consumption have been reported in CHA^(^^26^^–^^28^^)^. Whether or not these higher levels contribute towards a more favourable body composition among this population has yet to be established. Little evidence exists assessing the relationship between dietary protein intake, FM and FFM in CHA. We hypothesised that higher dietary protein intake would be associated with higher levels of FFM and lower BMI in a cross-sectional sample of CHA immigrants living in New York City.

## Materials and methods

### Study population

The Chinese American Cardiovascular Health Assessment (CHA CHA; ClinicalTrials.gov identifier: NCT00362128) was a cross-sectional epidemiological survey conducted on immigrant CHA (*n* 2071) living in New York City^(^^29^^–^^31^^)^. Data collection occurred from 2010 to 2011. Screening and recruitment have been previously reported^(^^31^^)^. During the study visit anthropometrics, sociodemographic, acculturation and behavioural information were collected. All participants provided written informed consent. The Institutional Review Board of the Albert Einstein College of Medicine and the New York Downtown Hospital (now New York Presbyterian Hospital of Lower Manhattan) approved the study.

### Study instruments and covariates

A Chinese-modified FFQ was used to assess reported total energy intake and macronutrient intake^(^^32^^)^. Participant data were excluded if total energy intake was ≤800 or ≥4000 kcal/d (≤3350 or ≥16 740 kJ/d) for males and ≤500 or ≥3500 kcal/d (≤2090 or ≥14 640 kJ/d) for females. Macronutrients were reported as a percentage of total energy intake. In addition, protein was expressed as g/kg BW (g protein/kg) and g/kg FFM (g protein/kg FFM). A validated Global Physical Activity Questionnaire (GPAQ) was used to measure physical activity (PA)^(^^33^^)^. Participant data were excluded if reported PA exceeded 960 min/d (16 h/d). Detailed descriptions of both the FFQ and GPAQ have previously been reported^(^^29^^,^^31^^)^. Questionnaires were presented in English with a Chinese translation below each question. The Stephenson Multigroup Acculturation Scale was used to measure acculturation, reported as ethnic society immersion and dominant society immersion^(^^34^^)^. Questionnaires were used to record self-reported characteristics: age, sex, income, smoking status, education level and years living in New York City. Participants were divided into age categories: young (21–44 years old), middle-aged (45–64 years old) and older adults (≥65 years old).

### Body composition

Height, BW and body composition were measured during the clinical visit by trained examiners. Body composition was assessed in light indoor clothing using a foot-to-foot bioelectrical impendence analysis (BIA) (Tanita TBF300a). The foot-to-foot BIA method accurately predicts FFM in healthy Asian individuals^(^^35^^)^. BMI, FMI and FFMI were calculated by dividing absolute FM and FFM by height in metres squared (kg/m^2^), respectively. FFM includes all non-fat tissue. FM percentage (FM%) and FFM percentage (FFM%) were calculated by dividing by BW. Participants were divided into the WHO Asian BMI categories: underweight (<18·5 kg/m^2^), normal weight (18·5–22·9 kg/m^2^), overweight (23·0–27·5 kg/m^2^) and obese (≥27·5 kg/m^2^)^(^^9^^)^.

### Statistical analysis

Mean and standard deviations were calculated for all variables except those in Table 3 where 95 % CI were calculated. Sex-specific distributions were calculated for the body composition variables, and *t* tests were used to calculate the differences between sexes. One-way ANOVA was used to determine the difference in body composition and dietary variables by WHO Asian BMI category and *post hoc* comparisons using Bonferroni's test. Two linear regression models were executed to determine associations between dependent variables (BW, BMI, FM, FMI, FM%, FFM, FFMI, FFM%) and the independent variables, protein intake as a percentage energy intake (%EI) and absolute intake in g/d. We decided to express protein intake as %EI in absolute intake, rather than relative to BW (g protein/kg BW). Evaluating protein intake relative to BW as a predictor variable and body composition (e.g. FM, FFM) as the outcome variable would put mass on both sides of the equation. Therefore, analysing protein relative to BW would inflate the variance explained between relative protein and body composition. Expressing protein intake relative to energy intake should offer a more accurate prediction of the contribution of protein to body composition. The first model included age, sex and PA (total moderate and total vigorous (min/week)). The second model includes age, sex, PA, acculturation (ethnic society immersion, dominant society immersion), total energy intake, sedentary time, smoking status, education, employment and income. To adjust for energy intake, we used the nutrient density method, which includes both the nutrient as a proportion of energy intake and total energy intake in the model as described in Willett *et al.*^(^^36^^)^. Multicollinearity diagnostics were run with no indication among independent variables (*r* < 0·80). However, there was strong correlation (*r* 0·89, variance inflation factor = 5·2) between energy intake and absolute protein intake. The level of significance was set at *P* < 0·05. All statistical analyses were conducted using SPSS version 23 (IBM).

## Results

The final sample included 1707 participants after excluding for PA values >960 min/d (*n* 299), and applying upper and lower total energy intake cut-offs (*n* 65). The mean age was 52·9 (sd 14·0) years, and 54·8 % were female (Table 1). The majority of participants had a high school (50·0 %) or college and above (35·1 %) education, and were employed (52·8 %); 8·7 % of participants reported an annual household income over $50 000. Table 2 presents the dietary and anthropometric data tabulated together and separated by sex. The mean energy from dietary carbohydrate, protein and fat was 53·0 (sd 8·8), 19·0 (sd 3·4) and 28·2 (sd 6·0) %, respectively. Comparing sexes, men consumed significantly greater total energy, g of carbohydrates, g of protein, g of fat, saturated fat, monounsaturated fat and polyunsaturated fat compared with women (*P* < 0·05). There was no significant difference in g of dietary fibre (*P* = 0·82) and relative protein (g protein/kg) (*P* = 0·06) between sexes. The acculturation variables (ethnic society immersion and dominant society immersion) were not associated with percentage protein (%EI). Absolute protein (g/d) was weakly associated with dominant society immersion (*r* 0·102; *P* < 0·01).

**Table 1.** Participant characteristics, Cardiovascular Health Assessment in Chinese Americans (CHA CHA) (Numbers of participants, percentages, mean values and standard deviations)

	*n*	%	Mean	sd
Overall	1707	100		
Age (years)	1707		52·9	14·0
Age category* (years)				
Young adult	472		35·6	6·9
Middle-age adult	862		54·2	5·3
Older adult	373		71·9	5·3
Female sex	935	54·8		
Smoking				
Current smoker	153	9·0		
Former smoker	394	23·1		
PA				
Total PA (min/week)	1707		375·2	334·1
Total moderate PA (min/week)	1707		344·4	306·2
Total vigorous PA (min/week)	1707		30·7	103·0
Sedentary time (min/d)	1707		328·6	174·7
Education				
No school and elementary	254	14·9		
High school	854	50·0		
College and above	599	35·1		
Income				
$0–9999	558	32·7		
$10 000–19 999	513	30·1		
$20 000–29 999	289	16·9		
$30 000–49 999	198	11·6		
$50 000+	149	8·7		
Employment				
Employed	902	52·8		
Unemployed	197	11·5		
Retired	418	24·5		
Housewife	190	11·1		
Time living in USA (years)	1707		13·3	10·4
Time living in New York City (years)	1707		12·5	10·3
Acculturation				
ESI†	1707		3·64	0·2
DSI†	1707		2·22	0·6

PA, physical activity; ESI, ethnic society immersion; DSI, dominant society immersion.

* Young adult, 21–44 years; middle-aged adult, 45–64 years; older adult, 65+ years.

† Scale range for ESI and DSI: 1–4.

**Table 2.** Energy intake and body composition by sex (Mean values and standard deviations)

	Total (*n* 1707)		Male (*n* 772)		Female (*n* 935)	
	Mean	sd	Mean	sd	Mean	sd	*P*: M *v*. F
Energy intake							<0·0001
kcal/d	1739·8	636·6	1900·5	651·4	1607·0	592·4	
kJ/d	7279·3	2663·5	7951·7	2725·5	6723·7	2478·6	
Carbohydrates (g/d)	227·7	84·3	249·6	87·2	209·5	77·2	<0·0001
Dietary fibre (g/d)	20·6	9·4	20·7	9·4	20·6	9·3	0·82
Protein (g/d)	83·3	35·8	90·4	37·6	77·4	33·3	<0·0001
Protein (g/kg BW per d)	1·34	0·59	1·31	0·56	1·36	0·61	0·06
Protein (g/kg FFM per d)	1·78	0·77	1·64	0·68	1·90	0·82	<0·0001
Fat (g/d)	55·5	26·0	58·8	27·3	52·7	24·6	<0·0001
SFA (g/d)	16·2	8·2	17·3	8·6	15·3	7·6	<0·0001
MUFA (g/d)	20·8	10·0	22·3	10·5	19·6	9·4	<0·0001
PUFA (g/d)	14·0	7·2	14·5	7·4	13·5	6·9	0·004
Height (cm)	162·3	8·5	168·5	6·6	157·3	6·2	<0·0001
BW (kg)	63·1	11·0	69·9	10·1	57·6	8·2	<0·0001
BMI (kg/m^2^)	23·9	3·2	24·6	3·2	23·3		<0·0001
Waist (cm)	83·1	9·1	86·3	8·4	80·4	8·9	<0·0001
FFM (kg)	47·5	8·7	55·4	6·4	41·0	3·4	<0·0001
FFM%	75·5	7·2	79·8	5·2	71·9	6·7	<0·0001
FFMI (kg/m^2^)	17·9	2·0	19·5	1·6	16·6	1·2	<0·0001
FM (kg)	15·6	5·8	14·5	5·2	16·6	6·0	<0·0001
FM%	24·5	7·3	20·2	5·2	28·1	6·9	<0·0001
FMI (kg/m^2^)	6·0	2·3	5·1	1·8	6·7	2·4	<0·0001

M *v*. F, male *v*. female; BW, body weight; FFM, fat-free mass; FFM%, fat-free mass as a percentage of total body weight; FFMI, fat-free mass index; FM, fat mass; FM%, fat mass as a percentage of total body weight; FMI, fat mass index.

The mean BMI for all participants was 23·9 (sd 3·2) kg/m^2^, which falls within the overweight category for WHO Asian BMI cut-offs^(^^9^^)^. FFM, FM% and FFMI were significantly higher in males compared with females (*P* < 0·0001), whereas the opposite was true regarding FM, FM% and FMI (*P* < 0·0001). Participants who were overweight or obese had higher FM whether expressed as kg, % or kg/m^2^ and higher absolute FFM (kg) but lower relative FFM (% or kg/m^2^) compared with participants classified as underweight or normal weight (Supplementary Table S1).

**Fig. 1.**
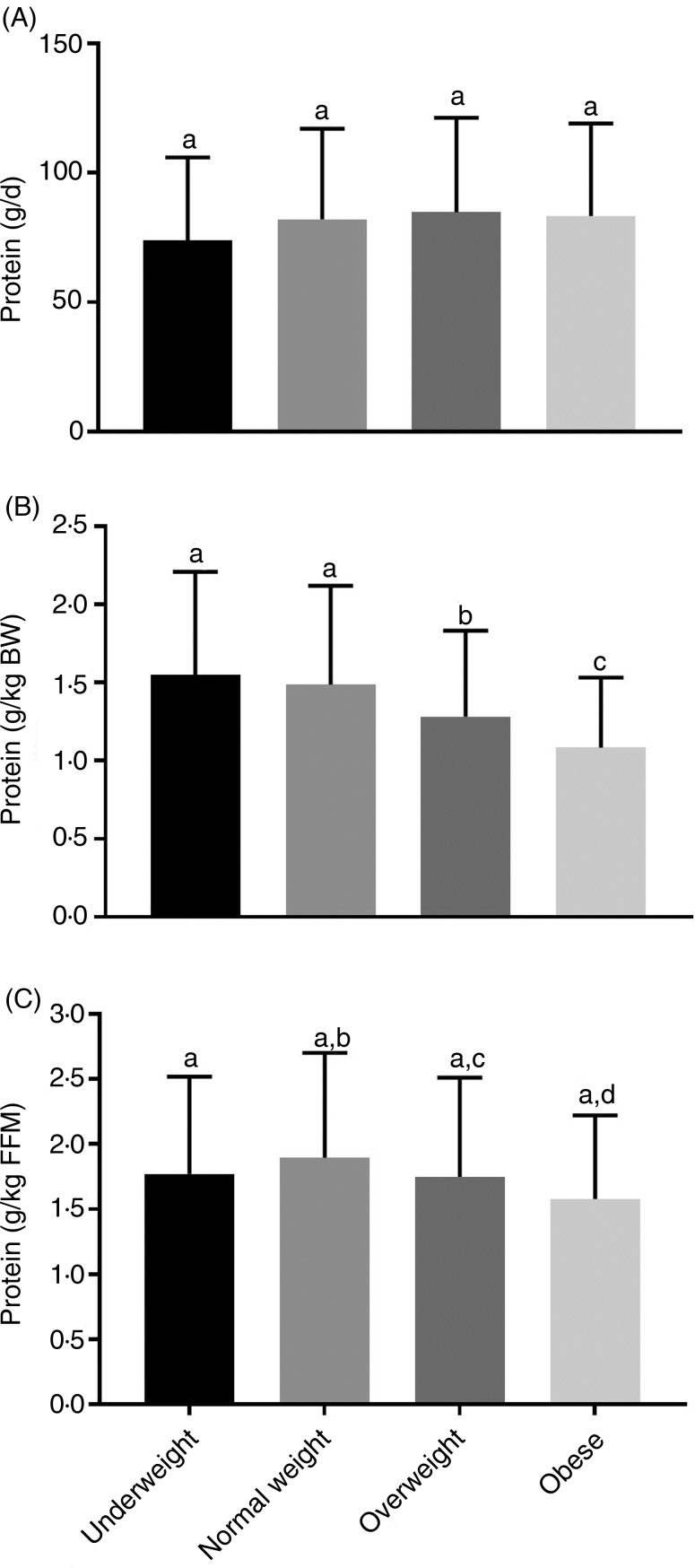
Dietary protein by BMI category. Relative protein intake (g protein/d) (A) was not significantly different across all BMI categories. Adjusting for body weight (BW) (B), underweight and normal-weight BMI categories had significantly greater relative dietary protein compared with overweight and obese BMI categories. Adjusting relative to fat-free mass (FFM) (C), normal-weight participants had significantly greater protein intake than overweight and obese participants. Underweight, *n* 59; normal weight, *n* 633; overweight, *n* 803; obese, *n* 214. Values are means, with standard deviations represented by vertical bars. ^a,b,c,d^ Mean values with unlike letters were significantly different (*P* < 0·05).

There was no significant difference among BMI categories for total energy intake, carbohydrates, protein and fat (Supplementary Table S1). There was no difference between relative protein (g/d) intake by BMI category (Fig. 1(A)), but participants in the underweight and normal-weight BMI categories consumed significantly more relative protein (g protein/kg) than those in the overweight and obese BMI categories (Fig. 1(B)). Adjusting for protein intake relative to FFM (g protein/kg FFM), participants in the underweight BMI category did not differ from all other groups. However, those in the normal-weight BMI category had significantly greater protein intake relative to FFM (g protein/kg FFM) compared with the overweight and obese categories (Fig. 1(C)). Protein intake stratified by age category indicates that younger adults consumed more absolute (g/d) and relative protein (g protein/kg and g protein/kg FFM) compared with middle-aged and older adults (Supplementary Table S2).

After adjusting for covariates, there was a significant positive association between protein (%EI) and BMI (*B* = 0·056, 95 % CI 0·017, 0·104; *P* = 0·005), FM (*B* = 0·106, 95 % CI 0·029, 0·184; *P* = 0·007), FM% (*B* = 0·112, 95 % CI 0·031, 0·194; *P* = 0·007) and FMI (*B* = 0·045, 95 % CI 0·016, 0·073; *P* = 0·002) (Table 3). There was a significant negative association between protein (%EI) and FFM% (*B* = −0·116, 95 % CI −0·196, −0·036; *P* = 0·004). However, absolute FFM and FFMI were not significantly associated with percentage protein (*B* = 0·026, 95 % CI −0·040, 0·092, *P* = 0·435; *B* = 0·02, 95 % CI −0·03, 0·037, *P* = 0·087). A similar relationship among all body composition variables occurred when protein was expressed in absolute terms (g/d), except there was no significant relationship between BW and absolute protein intake for both model 1 and model 2.

**Table 3.** Association between protein intake and body composition†

	Protein (%EI)				Protein (g/d)			
	*B*	95 % CI	*β*	*P*	*B*	95 % CI	*β*	*P*
Body weight (kg)								
Model 1	0·123	−0·005, 0·251	0·038	0·06	0·021	−0·007, 0·049	0·069	0·135
Model 2	0·132	0·004, 0·260	0·041	0·043*	0·023	−0·005, 0·050	0·073	0·109
BMI (kg/m^2^)								
Model 1	0·056	0·012, 0·100	0·060	0·012*	0·011	0·002, 0·021	0·123	0·022*
Model 2	0·060	0·017, 0·104	0·064	0·005**	0·011	0·002, 0·021	0·127	0·017*
FM (kg)								
Model 1	0·099	0·021, 0·176	0·059	0·013*	0·017	0·001, 0·034	0·108	0·042*
Model 2	0·106	0·029, 0·184	0·063	0·007**	0·018	0·002, 0·035	0·115	0·031*
FM%								
Model 1	0·103	0·021, 0·184	0·048	0·014*	0·019	0·002, 0·037	0·094	0·032*
Model 2	0·112	0·031, 0·194	0·052	0·007**	0·021	0·003, 0·038	0·101	0·022*
FMI (kg/m^2^)								
Model 1	0·041	0·012, 0·070	0·062	0·005**	0·008	0·001, 0·014	0·120	0·016*
Model 2	0·045	0·016, 0·073	0·067	0·002**	0·008	0·002, 0·014	0·126	0·011*
FFM (kg)								
Model 1	0·025	−0·041, 0·091	0·010	0·457	0·004	−0·01, 0·018	0·016	0·602
Model 2	0·026	−0·040, 0·092	0·010	0·435	0·004	−0·010, 0·018	0·017	0·559
FFM%								
Model 1	−0·106	−0·186, −0·026	−0·050	0·009**	−0·02	−0·037, −0·003	−0·10	0·021*
Model 2	−0·116	−0·196, −0·036	−0·055	0·004**	−0·022	−0·039, 0·004	−0·107	0·014*
FFMI (kg/m^2^)								
Model 1	0·017	−0·003, 0·037	0·028	0·01*	0·004	0·000, 0·008	0·069	0·074
Model 2	0·02	−0·03, 0·037	0·03	0·087	0·004	0·00, 0·08	0·070	0·072

%EI, percentage energy intake; *B*, unstandardised regression coefficient; *β*, standardised regression coefficient; FM, fat mass; FM%, fat mass as a percentage of total body weight; FMI, fat mass index; FFM, fat-free mass; FFM%, fat-free mass as a percentage of total body weight; FFMI, fat-free mass index; ESI, ethnic society immersion; DSI, dominant society immersion.

* *P* < 0·05, ** *P* < 0·001.

† Model 1: age, sex, physical activity; model 2: model 1 plus acculturation (ESI, DSI), total energy intake, sedentary time, smoking status, education, employment and income.

## Discussion

Our study, which examined the relationship between dietary protein intake and body composition measures (FM and FFM), found that in immigrant CHA (*n* 1707), higher dietary protein intake as a percentage of energy intake was associated with higher adiposity. Contrary to our hypothesis, our results demonstrate that percentage protein (%EI) was inversely associated with FFM%. However, absolute FFM and height-adjusted FFM (FFMI) were not associated with dietary protein intake. FFMI is a better indicator of non-fat tissue, accounting for differences in height within a population^(^^37^^)^. These results suggest that above-average intake of dietary protein as a percentage of total energy intake does not equate to higher levels of FFM in CHA.

In this cross-sectional analysis, higher protein intake was associated with higher BMI, FM and FMI after adjusting for covariates. Dietary protein, unlike dietary fat and carbohydrates, is not stored for the purpose of energy production. Under normal conditions the assimilation of amino acids from dietary protein into fatty acids to be stored in adipose tissue is unlikely. Under abnormal conditions, as with hyperenergetic feeding, protein intake (20–25 %EI) on the higher end of the acceptable macronutrient distribution range (AMDR) leads to minimal increases in FM among sedentary individuals^(^^38^^,^^39^^)^. Considering the majority of our population were active (>150 min/week of moderate-to-vigorous PA), the likelihood of energy from dietary protein contributing substantially to excess adiposity seems low.

Instead, the source of dietary protein (animal *v.* plant) may contribute to the positive association with BMI and FM. This sample may have consumed more animal protein, as we found protein intake to be positively associated with fat intake (*r* 0·64; *P* < 0·05) and inversely associated with carbohydrate intake (*r* −0·85; *P* < 0·01). Plant proteins are often lower in dietary fat and higher in carbohydrates, especially dietary fibre, which may modulate the diversity of gut microbiota affecting body composition^(^^40^^)^. Evidence from Chinese adults who underwent physical performance and appendicular skeletal muscle (ASM) assessment 4 years apart found no association between ASM and relative protein intake. However, participants in the highest quartile of relative vegetable protein lost significantly less ASM over 4 years^(^^41^^)^.

CHA may be consuming fewer lean sources of animal protein or consuming protein-containing dishes with added sources of fats (i.e. cooking oils). Animal sources provide both SFA and unsaturated fatty acids; we found protein intake to be significantly correlated with saturated fat (*r* 0·408; *P* < 0·0001) and monounsaturated fat (*r* 0·401; *P* < 0·0001). These positive relationships may result in the protein sources providing additional energy from dietary fat, which may lead to the propensity to gain weight.

The propensity of CHA to consume more dietary protein may be driven by acculturative stress. As opposed to a ‘Westernisation of diet’, increased consumption of traditional ‘festival foods’ (high in carbohydrates, animal protein, sugar and fat) among immigrants has been described as an explanatory factor in increased cardiometabolic risk in these populations^(^^42^^)^. Due to acculturative stress, immigrants may seek these foods as a source of comfort and maintenance of ethnic identity. However, adjusting for acculturation in our model was not associated with adiposity or FFM indices.

As aforementioned, US adults consume roughly 15 % of their energy from dietary protein^(^^25^^,^^26^^)^. Our results confirm previous evidence that CHA consume more protein compared with the general population. Recent data from the National Health and Nutrition Examination Survey (NHANES) 2011–2014 indicate that Asian adults (Chinese not specified) consumed roughly 17 % of energy intake from protein^(^^25^^)^. Nettleton *et al*.^(^^27^^)^ reported 17·7 % energy through a modified block-style 120-item FFQ intake from protein from a sample of CHA, and numbers adapted from Wong *et al*.^(^^28^^)^ were estimated to be around 19·2 % of energy intake from protein using three 24-h recalls in a sample of older CHA (>50 years old). In contrast, lower percentages have been reported in Chinese adults living in China. Stookey^(^^43^^)^ reported 12 % energy intake from protein using multiple 24-h recalls, and Lee *et al*.^(^^44^^)^ reported only 9 % energy intake from protein using an eighty-four-question FFQ in adults living in China.

Categorising the groups by BMI, absolute protein revealed no difference among the four BMI categories. Underweight and normal-weight groups consumed significantly more relative protein (g protein/kg) than adults in the overweight and obese groups. This may be attributed to under-reporting, common amongst adults with obesity^(^^45^^)^; however, the more likely reason is the higher body mass in both the overweight and obese groups. Further adjusting of protein relative to FFM (kg) abolished the difference between underweight, overweight and obese adults. However, we found that normal-weight participants consumed significantly more protein/kg FFM than both overweight and obese adults. Grouping by age category, younger adults in this CHA population consumed significantly more absolute (g/d) and relative protein (g protein/kg and g protein/kg FFM). Age-related declines in energy intake, and especially protein intake, are common. Recent research suggests that older adults may need to consume greater amounts (>0·8 g protein/kg) of protein to maintain and prevent muscle mass loss^(^^46^^,^^47^^)^.

The strengths of our study included a large sample, the inclusion of both sexes, and a comprehensive definition of body composition of an immigrant population. While our study found a positive relationship between dietary protein and body adiposity in CHA, there were some limitations. FFQ are useful for assessing diet–health relationships, providing information on long-term dietary intake and are commonly used in nutrition epidemiology. Unfortunately, they are not intended for the purpose of estimating energy intake as they may lack detail, are dependent on the number of items and may be affected by the recent diet^(^^48^^)^. Tseng & Hernández^(^^32^^)^ found a moderate correlation (*r* 0·30) between the FFQ used in this study and three 24 h recalls in a small sample of US Chinese women. Furthermore, the cross-sectional nature of the study only allows for conclusions between dietary protein and body composition to be associative and not causal. The relationship between body composition variables and absolute protein intake (g/d) should be interpreted with caution as evidence by collinearity between energy intake and absolute protein. Lastly, body composition assessment by BIA is not the ‘gold standard’ measure, nor does BIA provide a regional distribution of body adiposity. Using skin-fold callipers to assess body composition, Wang *et al*.^(^^11^^)^ found that that Asians (the majority Chinese) had more subcutaneous fat in the upper body (e.g. shoulders, biceps) and trunk than Caucasians. Regional changes in body composition may play a larger metabolic role rather than total body composition^(^^49^^)^. Despite these limitations, we believe that these results provide useful context between dietary intake and body composition among CHA immigrants.

In this population of CHA, dietary protein was associated with higher adiposity, specifically BMI and FMI, even after adjusting for covariates. Younger adults consumed significantly more absolute and relative protein compared with middle-aged and older adults. Contrary to our hypothesis, dietary protein was not associated with FFM and FFMI. Future research could employ more accurate measures of dietary intake (e.g. 24-h recalls) with ‘gold standard’ tools of body composition, or examine how sources of dietary protein may modify the effect of protein intake on body composition.

## Supplementary material

For supplementary material accompanying this paper visit https://doi.org/10.1017/jns.2018.31. click here to view supplementary material
