# Supplementary material for: A cross-sectional analysis of dietary protein intake and body composition among Chinese Americans
Source: J Nutr Sci. 2019 Jan 30;8:e4. doi: 10.1017/jns.2018.31 (PMC6360195; doi:10.1017/jns.2018.31)
Supplement: Supplementary file 1 [file S2048679018000319sup001.docx]

**Supplementary material**

|  | Underweight (n=59) | | Normal weight (n=631) | | Overweight (n=803) | | Obese (n=214) | |
| --- | --- | --- | --- | --- | --- | --- | --- | --- |
|  | Mean | SD | Mean | SD | Mean | SD | Mean | SD |
| Energy intake (kcal) | 1570.6 | 630.3 | 1716.6 | 619.2 | 1770.0 | 638.9 | 1745.0 | 673.5 |
| Carbohydrates (g) | 209.1 | 79.5 | 225.8 | 83.1 | 232.0 | 83.7 | 222.2 | 90.7 |
| Dietary fibre (g) | 17.5^a^ | 8.94 | 20.3 | 9.09 | 21.0^b^ | 9.60 | 20.9 | 9.32 |
| Protein (g) | 73.8 | 32.1 | 82.1 | 35.0 | 84.6 | 36.5 | 84.8 | 36.5 |
| Protein (g/kg BW) | 1.55^a^ | 0.65 | 1.49^a^ | 0.63 | 1.28^b^ | 0.55 | 1.08^c^ | 0.45 |
| Protein (g/kg FFM) | 1.77^a^ | 0.75 | 1.90^ab^ | 0.81 | 1.75^ac^ | 0.76 | 1.68^ad^ | 0.64 |
| Fat (g) | 49.5 | 29.4 | 54.7 | 25.1 | 56.0 | 26.3 | 57.3 | 26.9 |
| SFA (g) | 15.4 | 11.4 | 16.2 | 8.02 | 16.2 | 8.0 | 16.4 | 8.14 |
| MUFA (g) | 19.0 | 11.5 | 20.5 | 9.60 | 21.0 | 10.0 | 21.5 | 10.5 |
| PUFA (g) | 11.5^a^ | 5.95 | 13.6 | 6.82 | 14.3^b^ | 7.29 | 14.8^b^ | 7.75 |
|  |  |  |  |  |  |  |  |  |
| Ht (cm) | 163.9 | 11.3 | 161.5 | 7.83 | 162.7 | 8.57 | 162.9 | 9.07 |
| BW (kg) | 47.7^a^ | 6.69 | 55.3^b^ | 6.29 | 66.4^c^ | 7.94 | 78.2^d^ | 9.54 |
| BMI (kg/m^2^) | 17.4^a^ | 0.99 | 21.2^b^ | 1.18 | 25.0^c^ | 1.26 | 29.5^d^ | 1.95 |
| Waist (cm) | 69.5^a^ | 7.21 | 76.3^b^ | 6.03 | 86.1^c^ | 5.93 | 95.3^d^ | 7.0 |
| FFM (kg) | 41.8^a^ | 6.81 | 43.6^a^ | 6.50 | 49.2^b^ | 8.40 | 54.1^c^ | 10.0 |
| FFM (%) | 87.5^a^ | 4.53 | 78.8^b^ | 5.86 | 73.7^c^ | 6.06 | 68.9^d^ | 6.73 |
| FFMI (kg/m^2^) | 15.5^a^ | 1.01 | 16.6^b^ | 1.36 | 18.4^c^ | 1.65 | 20.2^d^ | 2.10 |
| FM (kg) | 5.87^a^ | 2.18 | 11.7^b^ | 3.36 | 17.2^c^ | 3.75 | 24.0^d^ | 4.94 |
| FM (%) | 12.4^a^ | 4.45 | 21.3^b^ | 6.29 | 26.3^c^ | 6.12 | 31.0^d^ | 6.70 |
| FMI (kg/m^2^) | 2.21^a^ | 0.82 | 4.51^b^ | 1.36 | 6.57^c^ | 1.62 | 9.16^d^ | 2.21 |

**Supplementary Table S1.** Dietary intake and body composition by BMI category

Data are reported as mean ± SD per day.

^a,b,c,d^ Different letters indicate significant, P<0.05.

Ht, height; BW, body weight; FFM, fat-free mass; FFMI, fat-free mass index; FM, fat mass; FMI, fat mass index.

**Supplementary Table S2.** Dietary intake by age category

|  | Young adults (n=472) | SD | Middle-age adults (n=862) | SD | Older adults (n=373) | SD |
| --- | --- | --- | --- | --- | --- | --- |
| Energy intake (kcal) | 1825.7^a^ | 644.5 | 1717.2^b^ | 636.3 | 1683.1^b^ | 617.7 |
| Carbohydrates (g) | 234.3 | 85.7 | 225.1 | 85.2 | 225.0 | 79.9 |
| Dietary fibre (g) | 19.56^a^ | 9.03 | 20.7 | 9.37 | 21.8^b^ | 9.67 |
| Protein (g) | 89.1^a^ | 38.1 | 82.1^b^ | 35.1 | 78.7^b^ | 33.8 |
| Protein (g/kg BW) | 1.47^a^ | 0.66 | 1.30^b^ | 0.56 | 1.29^b^ | 0.56 |
| Protein (g/kg FFM) | 1.89^a^ | 0.82 | 1.76^b^ | 0.74 | 1.75^b^ | 0.77 |
| Fat (g) | 59.2^a^ | 26.7 | 54.4^b^ | 25.5 | 53.2^b^ | 25.9 |
| SFA (g) | 17.7^a^ | 8.59 | 15.7^b^ | 7.95 | 15.3^b^ | 7.86 |
| MUFA (g) | 22.3^a^ | 10.2 | 20.4^b^ | 9.88 | 19.8^b^ | 9.81 |
| PUFA (g) | 14.3 | 7.13 | 13.9 | 7.05 | 13.8 | 7.44 |

Data are reported as mean ± SD per day.

^a,b^ Different letters indicate significant, P<0.05.

Young adult: 21 – 44 yr old; middle-aged: 45 – 64 yr old; older adult: >65 yr old; BW, body weight; FFM, fat-free mass.
